# External validation of the CURSI criteria (confusion, urea, respiratory rate and shock index) in adults hospitalised for community-acquired pneumonia

**DOI:** 10.1186/1471-2334-14-39

**Published:** 2014-01-22

**Authors:** Harald Nüllmann, Marc Andre Pflug, Thomas Wesemann, Hans-Jürgen Heppner, Ludger Pientka, Ulrich Thiem

**Affiliations:** 1 Department of Geriatrics, Marienhospital Herne, University of Bochum, Widumer Str. 8, Herne D-44627, Germany; 2 Department of Geriatrics, HELIOS Klinikum Schwelm, University of Witten/Herdecke, Dr.-Moeller-Str. 15, Schwelm D-58332, Germany; 3 Department of Medical Informatics, Statistics and Epidemiology, University of Bochum, Bochum D-44780, Germany

**Keywords:** Community-acquired pneumonia, Pneumonia severity, CURB-65, CRB-65, CURSI, Risk assessment, Mortality, Elderly, Inpatients

## Abstract

**Background:**

For patients hospitalised due to community-acquired pneumonia (CAP), mortality risk is usually estimated with prognostic scores such as CRB-65 or CURB-65. For elderly patients, a new score referred to as CURSI has been proposed which uses shock index (SI) instead of the blood pressure (B) and age (65) criteria. The new score has not been externally validated to date.

**Methods:**

We used data from a hospital-based CAP registry to compare the ability of CURSI, CURB-65 and CRB-65 to predict mortality at day 30 after hospital admission. Patients were stratified by score points as well as score-point-based risk categories, and mortality for each group was assessed. To compare test performance, receiver-operating characteristic (ROC) curves were constructed, and the areas under the curve (AUROC) were calculated with 95% confidence intervals (CI).

**Results:**

We analysed 553 inpatients (45% females, median age 78 years) hospitalised between 2005 and 2009 for CAP. Overall, mortality at day 30 was 11% (59/553). The study sample was characterised by advanced comorbidity (chronic heart failure: 22%, chronic kidney failure: 27%) and functional impairment (nursing home residency: 26%, dementia: 31%). All risk scores were significantly associated with 30-day mortality. The AUROC values with 95% CI using score points for risk prediction were as follows: 0.63 [0.56-0.71] for CRB-65, 0.68 [0.61-0.75] for CURB-65 and 0.68 [0.61-0.75] for CURSI. The CURSI-defined low-risk group (0 or 1 score point) had a higher mortality (8%) than the low-risk groups defined by CURB-65 and CRB-65 (4% and 3%, respectively). Lowering the cut-off for the CURSI-defined low-risk group (0 point only) would lower the mortality to 4%, making it comparable to the CURB-65-defined low-risk group.

**Conclusions:**

In our study, the CURSI-defined low-risk group had a higher 30-day mortality than the low-risk groups defined by CURB-65 and CRB-65. Lowering the cut-off value for the CURSI low-risk group would result in a mortality comparable to the CURB-65-defined low risk group. Even then, however, CURSI does not perform better than the established risk scores.

## Background

Community-acquired pneumonia (CAP) is a common and potentially life-threatening infectious disease. In Germany, the annual incidence of CAP is estimated at 400,000 to 680,000 infections per year [1]. In 2008, more than 200,000 CAP patients in Germany were treated in a hospital. Two-thirds were older than 70 years. In-hospital mortality was about 13% [2]. Apart from the threat to individuals, the high incidence of CAP also poses a considerable economic burden on the health care system: in 2008, the costs of pneumonia in Germany were estimated to reach a total of $1.5 billion [3]. Around $900 million was spent for inpatient treatment, which represents the major part (>90%) of the direct costs [3,4].

For this reason, it is highly important to identify every CAP patient‘s individual risk and severity of illness. To predict short-term mortality in CAP patients, national guidelines recommend the CURB-65 score [5,6]. In this simple score, one score point is awarded for each present risk factor (confusion, urea >7 mmol/L, respiratory rate ≥30/min, low systolic (<90 mm Hg) or diastolic (≤60 mm Hg) blood pressure, age ≥65 years). Increasing score points indicate increasing mortality [7]. CRB-65, a modification of CURB-65, is even easier to apply, since the measurement of blood urea is omitted. Thus, it consists of clinical parameters only that can be easily evaluated by clinical assessment, making it a useful tool in the ambulatory setting [7-10]. In line with the estimated risk of mortality, both CURB-65 and CRB-65 divide patients into three risk categories (‘low risk’, ‘intermediate risk’ and ‘high risk’) [7]. In recent years, both scores have been extensively validated [9,11-15].

Myint et al. [16] proposed a variant of CURB-65, referred to as CURSI, in which the age criterion is omitted and blood pressure is replaced by shock index, the ratio of heart rate to systolic blood pressure. One point is awarded when shock index values are higher than 1.0. Chronological age does not necessarily correlate with biological ageing, which may weaken the age criterion. The generally high prevalence of systolic hypertension in elderly patients may invalidate the blood pressure criterion [16]. As the shock index has been shown to be a predictor of mortality in CAP patients [17], it may prove to be a better marker for disease severity. In contrast to CRB-65 and CURB-65, CURSI divides patients into only two categories. The proposed risk score has not been externally validated to date. Therefore, the aim of our study was to compare the performance of the above-mentioned scores.

## Methods

### Setting and study population

This is a retrospective cohort study using data of the Marienhospital in Herne, Germany, a tertiary care university hospital with approximately 575 beds. It serves approximately 21,000 inpatients and 50,000 outpatients each year and provides unrestricted health care to the population.

Since 2005, all German hospitals providing acute medical care collect data on inpatients with CAP ≥18 years. This has been made compulsory for the purpose of external quality assurance. Variables included are: confusion due to pneumonia, respiratory rate and blood pressure upon admission to hospital, age, nursing home residency, immobility, mechanical ventilation and whether or not treatment was discontinued or not performed for various reasons.

For this study, data on all adult CAP inpatients treated at the Marienhospital Herne between 2005 and 2009 were extracted from the hospital’s CAP database. When reviewing the medical records, the diagnosis of CAP was considered to be confirmed if a patient presented at least one clinical symptom consistent with pneumonia (cough, dyspnoea, new or purulent sputum or fever) in combination with either a new pulmonary infiltrate or elevated laboratory values of inflammation within 48 hours of hospital admission in the absence of an alternative focus of infection. In this context, we also ascertained that the included patients did not suffer from acute exacerbation of chronic obstructive pulmonary disease, pneumonia due to other causes (for example aspiration, pulmonary embolism or bronchial stenosis) or immunodeficiency (e.g., due to HIV infection or chemotherapy). Laboratory values, specifically urea, were gathered from the central laboratory server, and additional diagnoses were obtained from the hospital information system. The patients’ pulse rate was assessed by means of either the admitting physician’s documentation or the electrocardiogram obtained at the time of admission. Information about the patients’ vital status and/or date of death was obtained from the responsible local register office (City of Herne Office of Statistics and Elections).

Patients who had discontinued or had not undergone treatment, those on mechanical ventilation upon admission or whose pulse rate values were missing, as well as patients referred from other hospitals were excluded.

### Sample size and statistical analysis

To calculate the sample size, we used a dichotomous risk factor (‘low risk’ versus ‘intermediate/high risk’) as defined for example by the CURB-65 score. Data of all adult inpatients that were treated at the hospital between 2001 and 2005 were available for sample size calculation. The data of patients aged ≥ 65 years of age have been reported elsewhere [18]. The data of adults aged < 65 years are unpublished. Using data of all adults, short-term mortality was strongly dependent on pneumonia severity, as assessed by CURB-65 [18]. On the basis of the dataset, we could expect a 30-day mortality of 5% in the low risk and at least 12% in the intermediate-/high-risk group. Using a significance level of 5% and a power of 80%, we calculated 248 cases per group with the chi-square test, or at least 500 patients in total.

Categorical variables are presented in absolute numbers and proportions, and continuous variables are presented with median, mean and range. All patients were stratified by score points as well as risk categories using the different severity scores CRB-65, CURB-65 and CURSI. The corresponding 30-day mortality was determined for each group. The association between categorical variables was assessed using chi-square test. We also calculated Odds Ratios (OR) with their corresponding 95% confidence interval (95% CI). To demonstrate the performance of the different scores in predicting death at day 30 after admission, receiver operating characteristic (ROC) curves were constructed for both score points and risk categories, respectively. The associated area under the ROC curve (AUROC) and corresponding 95% CI were calculated. For the identification of low-risk groups as defined by the different risk scores, we also calculated sensitivity, specificity and positive and negative likelihood ratios, all with 95% CI.

To calculate the sample size as well as sensitivity, specificity, likelihood ratios and the corresponding 95% CI, we used StatsDirect Software (version 2.7.9, 2012, StatsDirect Ltd., UK). All other analyses were performed with SPSS for Windows (version 21, 2012, IBM Inc., USA).

This study was approved by the ethics committee of the University of Bochum, Germany (registration no. 4376–12).

## Results

In total, 587 patients with CAP were identified by means of the CAP database. Patients who either discontinued or did not undergo treatment (18 patients), those on mechanical ventilation upon admission (six patients), patients referred from other hospitals (four patients) or whose pulse rate values were missing (six patients) were excluded, leaving 553 patients for analysis. The baseline characteristics are presented in Table 1.

**Table 1:** Characteristics of the study cohort (N = 553)

	**n**	**%**
a. Demographics		
Age ≥ 65 years	430	77.8
Sex (female)	246	44.5
b. Comorbidity		
Nursing home residency	145	26.2
Immobility	145	26.2
Congestive heart failure	121	21.9
Cerebrovascular disease	114	20.6
Dementia	170	30.7
Chronic lung disease	159	28.8
Diabetes mellitus	157	28.4
Chronic kidney disease	147	26.6
Neoplastic disease	35	6.3
c. Severity criteria		
Confusion	94	17.0
Urea >7 mmol/L	287	51.9
Respiratory rate ≥30/min	46	8.3
Blood pressure <90/≤60 mm Hg	133	24.1
Shock index >1.0	70	12.7
d. Outcome		
ICU* treatment during hospital stay	58	10.5
30-day mortality	59	10.7

*ICU: intensive care unit.

The patients’ median age was 78.2 years (mean 74.2; range 18.1–104.2). 78% were 65 or older. 246 patients (45%) were female. The collected data indicate a high prevalence of comorbidities, with over one-fifth of the patients suffering from chronic heart failure or kidney disease. Furthermore, a high proportion of patients (>25%) was immobile or living in nursing homes.

Among the 553 patients, 59 (10.7%) died within 30 days of admission. Age was strongly associated to mortality. Mortality at day 30 was 5% (6/123) in patients aged below 65 years, but 12% (53 / 430) in patients ≥ 65 years (p < 0.05; OR 2.74 [95% CI 1.15 – 6.54]). A comparable association was observed for shock index. In patients with a shock index below 1.0, mortality was 9% (44/483), whereas patients with a shock index ≥ 1.0 had a mortality of 21% (15/70; p < 0.01; OR 2.72 [95% CI 1.42 – 5.21]).

Table 2 shows 30-day mortality according to the pneumonia severity scores CRB-65, CURB-65 and CURSI, ordered by score points and risk categories, respectively. Overall, mortality increased significantly with increasing score points and risk categories of each severity score.

**Table 2:** 30-day mortality stratified by score points and risk categories

**Severity score**	**Score points**	**No. of patients**	**Mortality**	
		**N = 553**	**n**	**%**
CRB-65^1^	0	87	3	3.4
	1	271	24	8.9
	2	155	24	15.5
	3	38	8	21.1
	4	2	0	0
CURB-65^2^	0	73	2	2.7
	1	138	7	5.1
	2	200	23	11.5
	3	116	19	16.4
	4	26	8	30.8
	5	0	0	0
CURSI^3^	0	195	8	4.1
	1	240	26	10.8
	2	98	16	16.3
	3	19	8	42.1
	4	1	1	100
**Severity score**	**Risk category**	**No. of patients**	**Mortality**	
		**N = 553**	**n**	**%**
CRB-65^4^	Low risk	87	3	3.4
	Intermediate risk	426	48	11.3
	High risk	40	8	20.0
CURB-65^5^	Low risk	211	9	4.3
	Intermediate risk	200	23	11.5
	High risk	142	27	19.0
CURSI^6^	Low risk	435	34	7.8
	High risk	118	25	21.2

Definition of risk categories.

CRB-65: low risk = 0 points; intermediate risk = 1–2 points; high risk = 3–4 points.

CURB-65: low risk = 0–1 points; intermediate risk = 2 points; high risk = 3–5 points.

CURSI: low risk = 0–1 points; high risk = 2–4 points.

*P-values according to chi-square test:*^1^p = 0.007; ^2^p < 0.001; ^3^p < 0.001; ^4^p = 0.014; ^5^p < 0.001; ^6^p < 0.001.

The number of patients in the low-risk groups was lowest for CRB-65 (87 patients) compared to both urea-based severity scores (CURB-65: 211 patients; CURSI: 435 patients). Thirty-day mortality in the low-risk groups was highest for the CURSI (34 patients, 8%). This high mortality could be reduced at the expense of group size by using an alternative cut-off, i.e., defining only patients scoring 0 points as low-risk patients (instead of 0 or 1 point). In this case, 195 patients would remain in the CURSI-defined low-risk group. The resulting mortality in this group (8 patients, 4%) would be comparable to that of the low-risk group defined by CURB-65 (9 patients, 4%).

**Figure 1:**
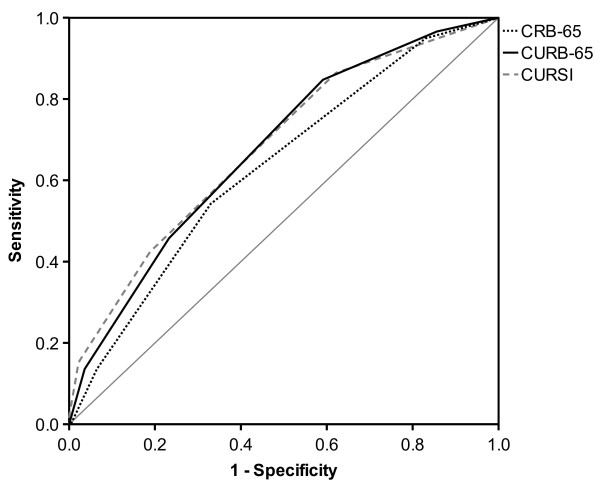
Receiver operating characteristic (ROC) curves for score points.

**Figure 2:**
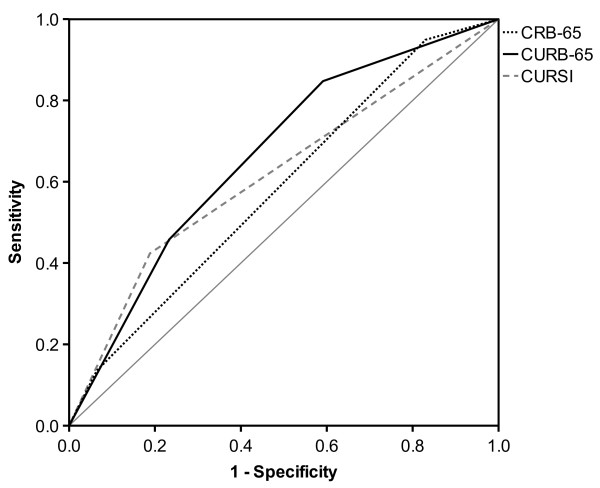
Receiver operating characteristic (ROC) curves for risk categories.

These findings are also reflected by ROC analysis, as shown in Table 3 and Figures 1 and 2. In terms of risk categories, CURSI performed worse (AUROC value: 0.62) than the CURB-65 (0.67), whereas for score points, AUROC values of CURSI and CURB-65 proved to be comparable. The AUROC of the CRB-65 was lowest in both cases.

**Table 3:** Area under the receiver operating characteristic curve (AUROC) for CRB-65, CURB-65 and CURSI

**Severity score**	**AUROC**	**95% CI**
*Score points*		
CRB-65	0.633	0.560–0.705
CURB-65	0.676	0.607–0.746
CURSI	0.682	0.609–0.754
*Risk categories*		
CRB-65	0.585	0.511–0.659
CURB-65	0.665	0.595–0.735
CURSI	0.618	0.536–0.699

Definition of risk categories.

CRB-65: low risk = 0 points; intermediate risk = 1–2 points; high risk = 3–4 points.

CURB-65: low risk = 0–1 points; intermediate risk = 2 points; high risk = 3–5 points.

CURSI: low risk = 0–1 points; high risk = 2–4 points.

When patients were dichotomised into a low-risk and a non-low-risk group, sensitivity, specificity and corresponding 95% CI were as follows: for CRB-65 95% [86%–99%] and 17% [14%–21%], for CURB-65 85% [73%–93%] and 41% [37%–45%] and for CURSI 42% [30%–56%] and 81% [77%–85%], respectively. Hence, the CURSI had the lowest sensitivity with a resulting higher mortality in the low-risk group. Using the alternative cut-off (0 points), values for the CURSI-defined risk groups would change to 86% [75%–94%] and 38% [34%–42%]. This is similar to the characteristics of CURB-65, as mentioned above.

The following positive likelihood ratios were found: for CRB-65 1.14 [1.07-1.23], for CURB-65 1.43 [1.26-1.63], and for CURSI 2.25 [1.59-3.19]. The corresponding negative likelihood ratios for CRB-65, CURB-65 and CURSI were: 0.30 [0.10-0.92], 0.37 [0.20-0.69], and 0.71 [0.57-0.89].

## Discussion

The aim of our study was to assess the performance of the CAP severity score CURSI [16] in predicting short-term mortality and to compare it to the established scores CRB-65 and CURB-65. Our findings show that increasing score points and risk categories of CURSI are associated with increasing 30-day mortality in CAP patients. However, CURSI does not outperform the other scores, and has some limitations.

One of the most important steps in managing CAP involves deciding whether a patient can safely be regarded as being at low risk of mortality. This would offer the perspective to align several management decisions to risk, with hospital admission being the most important decision. Admission to hospital multiplies the treatment costs, harbours the danger of hospital-acquired infections and is associated with a slower return to work and usual activities [19-22]. Therefore, our analysis focused on the ability of the severity scores to identify patients at low risk of mortality, or in other words on the sensitivity of the severity scores used. In comparison to CRB-65 and CURB-65 (sensitivity about 95% and 85%, respectively), CURSI had a markedly lower sensitivity (around 42%). The 95% CI showed no overlap between CRB-65 or CURB-65 and CURSI. In our view, this finding indicates a clinically important difference in sensitivity between CBR-65 or CURB-65 and CURSI that is unlikely to be explained by chance alone. The mentioned finding is also reflected by the negative likelihood ratios found. The negative likelihood ratios show that being identified as low risk by CRB-65 or CURB-65 indicates a much lower chance for mortality at day 30 than being defined as low risk by CURSI.

Compared to CURB-65, CURSI identifies twice as many patients as being at low risk. However, this possible advantage is nullified by the high mortality in this group. This limitation could only be avoided by lowering the proposed cut-off [16]. The resulting values (sensitivity, specificity, number of patients and mortality in the low-risk group) would be very similar to those of CURB-65. However, a modification of the CURSI-defined low-risk group would require further validation studies. By contrast, CRB-65 and CURB-65 have already been well validated in recent years [9,11-15].

Two further aspects deserve consideration. The risk categories as proposed for the CURSI do only differentiate between a low risk and a high risk group. By contrast, CRB-65 and CURB-65 based categories also define an intermediate risk group. Current guidelines [23,24] do not recommend basing the indication for ICU admission on severity scores, like CRB-65 or CURB-65. However, the differentiation of patients not being low risk may allow a further differentiation of treatment intensities, for example intensity of clinical monitoring. Further, in our opinion, replacing the criteria age and blood pressure of CURB-65 by shock index in CURSI offers no practical advantage. Age can be assessed easily in nearly all patients. Blood pressure is also part of the shock index and thus has to be measured in both cases.

The motivation for developing CURSI was the assumed inaccuracy of the criteria age and blood pressure of CURB-65 with reference to the high prevalence of hypertension in elderly patients and the low correlation between chronological and biological age [16]. The shock index was purported to be a better marker for pneumonia severity and had already been shown to correlate with mortality in CAP patients [17]. We can confirm this observation with our data. However, we can also confirm that age is a strong predictor of short-term mortality, which has already been shown repeatedly [15,25-27]. On this basis, a simple, yet likely explanation why CURSI is not able to outperform CRB-65 or CURB-65 would be that it only substitutes one strong predictor variable (age) with another (shock index).

The test performance of CURB-65 and CRB-65 found in our analysis is comparable to other studies. In their analysis describing and evaluating the CURB-65, Lim et al. [7] reported a sensitivity of 93% and a specificity of 49% for the identification of the low risk group. The performance of the CRB-65 was worse (77% and 64% sensitivity and specificity, respectively), but still comparable. Likewise, several other studies found sensitivity and specificity for CURB-65 to be between 80%-90% and 50%-60%, respectively [9,13,28]. In the original CURSI study [16], Myint et al. reported only 60% and 75% for sensitivity and specificity for the CURB-65. For CURSI, the figures were 61% and 72%, which is somewhat better than the estimates we derived from our analysis. However, as it seems, the equivalent results of Myint et al. for CURSI and CURB-65 are caused by the relatively weak performance of the CURB-65 rather than a good performance of the CURSI.

The AUROC of the CRB-65 was lower compared to the other severity scores. This can be explained by the omission of the urea criterion, which has been shown to be significantly associated with mortality in CAP patients [7,29-31]. Furthermore, CRB-65 identified fewer patients at low risk than CURB-65 (87 vs. 211), but showed better sensitivity with a resulting even slightly lower mortality in the low-risk group. These findings are consistent with several other studies [7,9,12,28,32]. CRB-65 is recommended for use in ambulatory care [6,23,24], as CURB-65 requires the blood urea value, which may not be available in all cases or might delay decision-making. In a large prospective German study including inpatients and outpatients with CAP [33], blood urea was available for fewer than half of the outpatients included in the study.

Our study has limitations. One limitation is the retrospective design and the use of registry data for analysis. However, we do not believe that this affects the validity of our findings. The registry was established for the purpose of quality assurance and contains reliable and complete information on all variables of the CRB-65. Data are entered into the database in time, i.e. during hospital stay of the patient or immediately after discharge. The corresponding blood urea values could be retrieved from the central laboratory server without any missings. Pulse rate was clearly documented on admission in all but six cases (1.1% of all cases). Reliable information on 30-day mortality as the primary endpoint was provided for all patients by the relevant local register office. With this, we are convinced that the data for the variables of interest, namely the three severity scores used and mortality as the primary endpoint, are valid. Another limitation is that only inpatients were included. Therefore, no statement can be made as to whether patients identified as being at low risk can be safely treated as outpatients. This hypothesis demands testing in prospective, randomised controlled trials. Finally, we present data from a single centre only. In terms of CRB-65 and CURB-65 performance, our results are in line with several other studies. However, as characteristics of hospitals and populations served can differ widely, generalisability of our findings is certainly limited.

## Conclusions

The risk categorisation based on the new CAP severity score CURSI was not suitable for predicting low risk of death in our analysis. By lowering the cut-off for the low-risk group, CURSI could match CURB-65 in terms of identifying low-risk patients. However, using CURSI instead of CURB-65 provides no practical advantage, and CURB-65 is much better validated and already implemented in current guidelines.

## Competing interests

The authors declare that they have no competing interests.

## Authors’ contributions

HN analysed and interpreted the data, performed the literature review and drafted the manuscript. MP and TW contributed to the design of the study, collected the data and supported data analysis and interpretation. HJH and LP designed the study and interpreted the data. UT designed the study, contributed to the collection of data, performed analysis and interpretation of the data and assisted in preparing the first draft of the manuscript. All authors read and approved the final version of the manuscript.

## Pre-publication history

The pre-publication history for this paper can be accessed here:

http://www.biomedcentral.com/1471-2334/14/39/prepub
